# Reactive Oxygen Species Induce Fatty Liver and Ischemia-Reperfusion Injury by Promoting Inflammation and Cell Death

**DOI:** 10.3389/fimmu.2022.870239

**Published:** 2022-04-29

**Authors:** Shen-ping Tang, Xin-li Mao, Ya-hong Chen, Ling-ling Yan, Li-ping Ye, Shao-wei Li

**Affiliations:** ^1^ Taizhou Hospital of Zhejiang Province Affiliated to Wenzhou Medical University, Linhai, China; ^2^ Key Laboratory of Minimally Invasive Techniques & Rapid Rehabilitation of Digestive System Tumor of Zhejiang Province, Taizhou Hospital Affiliated to Wenzhou Medical University, Linhai, China; ^3^ Department of Gastroenterology, Taizhou Hospital of Zhejiang Province affiliated to Wenzhou Medical University, Linhai, China; ^4^ Institute of Digestive Disease, Taizhou Hospital of Zhejiang Province Affiliated to Wenzhou Medical University, Linhai, China; ^5^ Health Management Center, Taizhou Hospital of Zhejiang Province Affiliated to Wenzhou Medical University, Linhai, China

**Keywords:** ROS, hepatic ischemia-reperfusion, fatty liver, apoptosis, inflammation

## Abstract

Liver transplantation is the ultimate method for treating end-stage liver disease. With the increasing prevalence of obesity, the number of patients with non-alcoholic fatty liver, a common cause of chronic liver disease, is on the rise and may become the main cause of liver transplantation in the future. With the increasing gap between the number of donor livers and patients waiting for liver transplantation and the increasing prevalence of non-alcoholic fatty liver, the proportion of steatosis livers among non-standard donor organs is also increasing. Ischemia-reperfusion injury has historically been the focus of attention in the liver transplantation process, and severe ischemia-reperfusion injury leads to adverse outcomes of liver transplantation. Studies have shown that the production of reactive oxygen species and subsequent oxidative stress play a key role in the pathogenesis of hepatic ischemia and reperfusion injury and non-alcoholic fatty liver. Furthermore, the sensitivity of fatty liver transplantation to ischemia-reperfusion injury has been suggested to be related to the production of reactive oxygen species (ROS) and oxidative stress. In ischemia-reperfusion injury, Kupffer cell and macrophage activation along with mitochondrial damage and the xanthine/xanthine oxidase system promote marked reactive oxygen species production and the inflammatory response and apoptosis, resulting in liver tissue injury. The increased levels of ROS and lipid peroxidation products, vicious circle of ROS and oxidative stress along with mitochondrial dysfunction promoted the progress of non-alcoholic fatty liver. In contrast to the non-fatty liver, a non-alcoholic fatty liver produces more reactive oxygen species and suffers more serious oxidative stress when subjected to ischemia-reperfusion injury. We herein review the effects of reactive oxygen species on ischemia-reperfusion injury and non-alcoholic fatty liver injury as well as highlight several treatment approaches.

## Introduction

When the liver disease reaches terminal failure, the last resort is liver transplant. With the increasing global prevalence of obesity, type 2 diabetes, and metabolic syndrome, more patients than ever before are being diagnosed with non-alcoholic fatty liver disease (NAFLD) and reaching the terminal stage of their disease, it is reported that the global prevalence of NAFLD is about 25% (1). Patients with NAFLD may thus account for the greatest proportion of liver transplant candidates in the future (2). Over the past 10 years, the gap between the number of patients awaiting liver transplantation and the number of donor livers has increased, and the possibility of receiving a non-standard donor organ, including a fatty liver, has increased (3).Steatosis is considered when evaluating donor livers,which may affect transplant outcomes and is approximately 30% in deceased organ contributors and approximately 20% in living donors. In previous studies, the detection rate of NAFLD in living donor livers was between 14.5-53% (4). An evaluation of potential donors for living donor liver transplantation (LDLT) programs in Malaysia showed that NAFLD and obesity were significant reasons for low donor utilization (5). Severe steatosis liver (>60%) excludes donor liver range, while the use of moderate steatosis (30-60%) remains controversial (6, 7).

Ischemia and reperfusion (IR) injury is inevitable in cases of liver transplantation. IR injury involves various mechanisms, including macrophage polarization, necroptosis, reactive oxygen species (ROS) production, and oxidative stress (8). During liver reperfusion, the activation of Kupffer cells (KCs) and macrophages, mitochondrial damage, and large amounts of xanthine and hypoxanthine due to increased ATP consumption during ischemia promote the production of a large quantity of ROS (9). Excessive ROS and the consumption of endogenous antioxidants lead to redox imbalance, which causes oxidative stress (10). ROS combine with intracellular macromolecules to damage organelles, which eventually leads to cell damage and even apoptosis (11). Active oxygen damages lipids, resulting in the production of lipid peroxides, destruction of cell membrane permeability, apoptosis, the production of inflammatory factors, and the induction of inflammatory reactions (12). Reactive oxygen can also cooperate with Ca^2+^ to promote the opening of mitochondrial membrane permeability transition pore (MPTP), damage to mitochondria, release of cytochrome C, formation of apoptotic bodies, and promotion of cell apoptosis (13). Furthermore, cells that are damaged by ROS release damage-related model molecules (DAMPs), which combine with TLR4 or TLR9 on KCs to activate the NF-κB signaling pathway and thus generate more ROS to amplify the inflammatory response (14, 15).

When fatty livers are subjected to IR, they generate more ROS than non-fatty livers, the lipid peroxidation reaction is heavier, the antioxidant capacity is lower, and the mitochondria are more vulnerable to damage, so the resulting tissue damage is more serious. Such increased sensitivity and reduced tolerance of NAFLD to IR have increased the difficulty of treatment.

We herein review the role of ROS and its influence on fatty liver transplantation.

## The Production of ROS in IR

**Figure 1:**
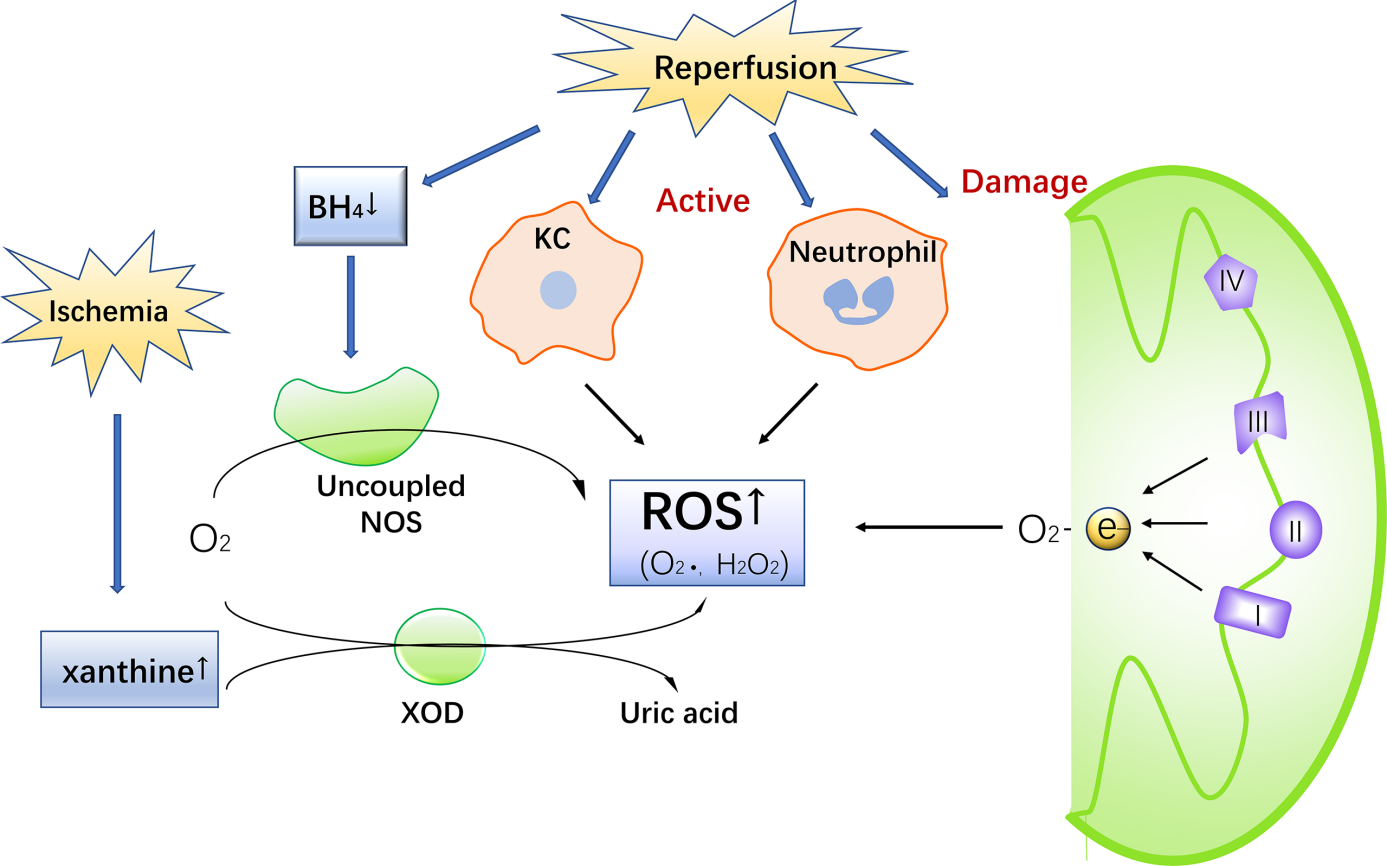
The Production of ROS during IR injury. Ischemia leads to an increase in xanthine. Reperfusion damages mitochondria, activates Kupffer cells and neutrophils, consumes BH4 and uncouples NOS, promotes ROS production, and simultaneous increase of xanthine in the catalysis of xanthine oxidase to produce ROS.

During IR, ROS are produced by a variety of pathways, causing oxidative stress and damage. ROS in IR come are generated by a number of different sources, including the mitochondrial electron transport chain, xanthine oxidase, NADPH oxidase, and uncoupling nitric oxide synthase (NOS) (16) (**Figure 1**).

### Mitochondrial Electron Transport Chain

ROS are mainly produced in the respiratory chain complex on the mitochondrial inner membrane during mitochondrial metabolism (9, 11). Under normal circumstances, mitochondria produce a small amount of ROS (17). However, when subjected to ischemia, a large quantity is produced in order to maintain the redox balance, leading to the consumption of endogenous antioxidants and the expression of antioxidant enzymes. When the damage is severe or sustained, the ROS-scavenging capacity of the antioxidant system is not sufficient to remove the increased amount of ROS, leading to oxidative stress, inflammation, cell death, and organ failure (10, 18). In the ischemic state, prolonged tissue hypoxia and the consequent increase in ATP depletion often lead to cell damage. Therefore, the cell metabolism pattern becomes anaerobic, leading to the accumulation of lactic acid, a decrease in intracellular pH, and disorders of cytoplasmic ions (19, 20). Taken together, these events can damage mitochondrial macromolecules, such as DNA and protein, and the damaged mitochondria gradually produce more ROS, creating a vicious cycle of ROS production and mitochondrial damage (11).

### Xanthine Oxidase

Xanthine oxidizing reductase (XOR) can catalyze the conversion of hypoxanthine to xanthine, which is then converted to uric acid. It exists in the form of XO and XDH, and these two subtypes can be converted to each other (21).The xanthine/xanthine oxidase (XOD) system is an important source of ROS in hepatocytes. During the ischemic period, xanthine dehydrogenase (XDH) is transformed into XOD, which produces oxygen-free radicals. At the same time, xanthine, the substrate of XOD, is also accumulating. During the reperfusion period, XOD is transformed to ROS (22). The use of allopurinol, a type of XOD inhibitor, can inhibit ROS production and reduce liver IR injury (23).

### NADPH Oxidase

The Nox/Duox family in NADHP oxidase, including NOX1-5, DUOX1 and DUOX2, is involved in the production of ROS (24). The increase in ROS after reperfusion is an important part of IR injury (25). During reperfusion, the depletion of superoxide dismutase (SOD) and glutathione (GSH) peroxidase and reduced GSH promote oxidative stress (26). Reperfusion can be divided into two stages. The initial stage is 0.5-4 h after reperfusion, and the late stage is 6-24 h after reperfusion (18). In the initial stage of reperfusion, KCs are activated to express Nox2 and its subunits, forming complexes on the cell membrane, catalyzing the reduction of oxygen and producing ROS, thus causing oxidative stress (24, 27, 28). At the same time, T lymphocytes secrete interferon-α (IFN-α) to promote the activation of KCs (9). In the late stage of reperfusion, oxidants derived from extrahepatic cells mediate inflammatory damage (29). Under constant stimulation, neutrophils continuously activate the NADPH oxidase complex, producing a large number of ROS (30). Meanwhile, neutrophils infiltrate, secreting pro-inflammatory factors, such as TNF and platelet-activating factor. TNF induces the expression of adhesion molecules in vascular endothelial cells to recruit neutrophils. Platelet activators are involved in the activation of ROS production by neutrophils (9). When neutrophils attack liver cells, they produce hydrogen peroxide and hypochlorous acid, which are cytotoxic and damage liver cells (31).

### NOS

In addition, during IR, the uncoupling of NOS also produces ROS (32). Coupled NOS oxidized L-arginine to nitric oxide (NO). NO attenuates IR injury through antioxidation and the inhibition of inflammatory cell migration, as well as to reductive superoxide (33, 34). IR reduces the content of BH4, a cofactor of nitric oxide synthase, leading to the uncoupling of NOS, thereby resulting in the production of ROS (33).

## ROS Causes Damage to Liver During Ischemia-Reperfusion

**Figure 2:**
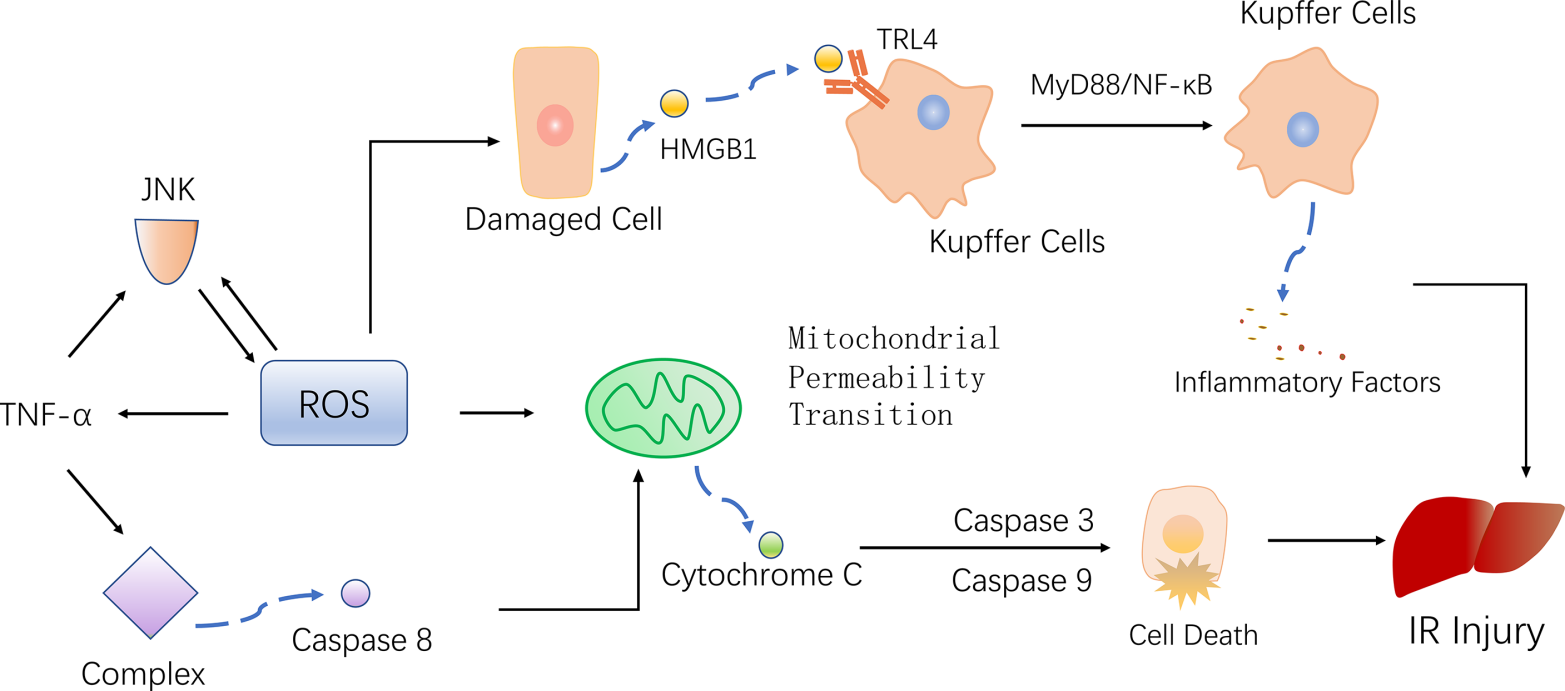
ROS mediates inflammation, mitochondrial damage, and cell death, ultimately promoting liver damage. KCs recognize HMGB1, promote the inflammatory response through the TRL4-MyD88-NF-κB pathway. At the same time, TNF-α activated by ROS, phosphorylating JNK and further producing ROS. Activated TNF-α also form complexes that activate caspase 8, leading to mitochondrial permeability transition (MTP) and finally apoptosis.

Under normal circumstances, the production and removal of ROS in the body are in a dynamic equilibrium state, and there is no harmful effect on the body. However, when a cell encounters IR, in order to adapt to or resist the stimulus, the cell produce a large amount of highly active ROS. On one hand, ROS activate the antioxidant system, but on the other hand, they attack and damage endogenous antioxidants (35). The excessive production of ROS and weakened antioxidant capacity in cells lead to an imbalance between the production and removal of ROS, resulting in oxidative stress and thereby promoting inflammation, cell apoptosis and mitochondrial damage (36, 37) (**Figure 2**).

### ROS Promote Inflammation

During reperfusion, a large amount of ROS is produced, which causes oxidative stress and cell and even tissue damage. ROS activates KCs and macrophages, subsequently the activated cells release a large number of pro-inflammatory cytokines, including TNFa, IL-1b, etc., which promote the inflammatory response to damage hepatocyte damage and accelerate the progression of IR damage (38). In the initial stage of reperfusion, KCs recognize and activate endogenous damage-related model molecules (DAMPs) released by damaged cells, which recruit monocytes and neutrophils to synergistically induce inflammation. Common DAMPs include high mobility group box-1 protein (HMGB1), DNA, and ATP (14). HMGB1 is a DNA-binding protein. Normally, HMGB1 is expressed in organs and tissues, but when IR occurs, HMGB1 is released from the damaged nucleus and into the blood under the promotion of ROS, leading to cell death (39). HMGB1 binds to toll-like receptor 4 (TLR4) on KCs and activates the NF-κB signaling pathway, leading to the massive release of proinflammatory factors (IL-1, IL-2, IL-3, IL-6, IL-8, TNF-α) and subsequent promotion of inflammatory reactions (14, 39, 40). DAMPs can also activate TLR9 on KCs, enhancing the production of TLR9-dependent ROS and inflammatory mediators and promoting inflammation. TLR9-knockout mice show a reduced inflammatory cytokine production and liver damage compared with wild-type mice (15, 41).

### ROS Induce Mitochondrial Damage

ROS attacks mitochondria, leading to mitochondrial damage. The mitochondrial apoptosis pathway is one of the main apoptotic pathways, and the continuous opening of the mitochondrial membrane permeability transition pore (MPTP) is an important mechanism of mitochondrial function damage (42). The massive generation of ROS and Ca^2+^ overload caused by IR regulates the opening of the MPTP, resulting in the irreversible transformation of mitochondrial permeability transition (MPT). As a result, the electron transport chain is uncoupled, damaging the mitochondrial membrane. Cytochrome C is released through the channel formed by Bax to produce apoptotic bodies, which mediate endogenous cell apoptosis (13, 43). Cytochrome C released into the cell forms a complex with apoptosis activator 1, activating caspase-9 and caspase-3. This activated caspase-3 damages DNA repair enzymes, aggravating cell DNA damage and leading to cell apoptosis (44). Sirtuin1 (SIRT1) is an NAD+-dependent class III protein deacetylase that regulates hepatic lipid metabolism, systemic inflammatory status and autophagy through mitofusin2 (MFN2) (45). In Overexpression of STRT1 and (MFN2) in mice promotes autophagy and prevents mitochondrial dysfunction (46).

### IR Induce Apoptosis

During IR, multiple types of cells such as Kupffer cells and lymphocytes release TNF-α, which induces NOS and chemotaxis of leukocytes (47). When TNF-α binds to a specific receptor, the death signaling pathway, which depends on c-Jun N-terminal kinase (JNK) phosphorylation, is activated, inducing the production of ROS in mitochondria. Aggravated oxidative stress further stabilizes the JNK phosphorylation level, resulting in MPT and consequent cell death (16). Under the continuous stimulation of TNF-α, TNF-α receptor-associated protein with death domain(TRADD) bind to the Fas-associated protein with death domain(FADD), summon and activate Caspase 8, which leads to changes in mitochondrial permeability and initiates the process of apoptosis (48–50). TNF-α can also increase the expression of NF-κB, thereby promoting the production of ROS and causing damage as well as gathering CD4+ T cells, promoting the secretion of colony-stimulating factors (IFN-γ and TNF-β), and accelerating cell apoptosis during IR (9).

In IR, autophagy has both damaging and protective effects. Proper mitochondrial autophagy during the ischemic stage can clear away damaged mitochondria and reduce subsequent injury. However, during the reperfusion stage, ROS levels are significantly increased, the MPTP remains open, and the PINK1/Parkin pathway is activated, mediating mitochondrial autophagy and concentrated injury (51).

## ROS and Fatty Liver Transplantation

**Figure 3:**
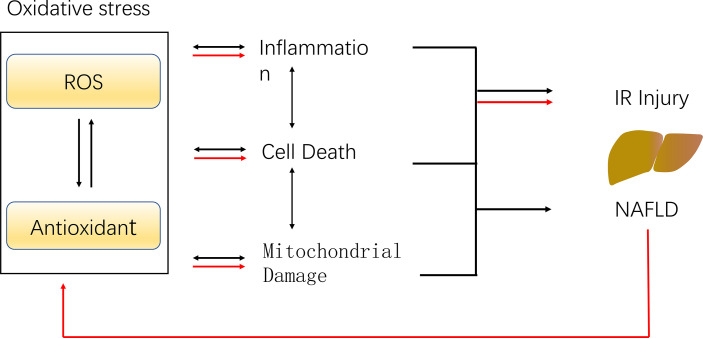
The potential mechanism of IR injury and NAFLD caused by ROS. ROS leads to the inactivation of antioxidants, which reduce the scavenging of ROS, and the imbalance of the ROS and antioxidant results in oxidative stress. Oxidative stress promotes inflammation, mitochondrial damage as well as cell death, leading to hepatic ischemia-reperfusion injury and NAFLD (black arrows). The presence of NAFLD resulted in excessive ROS generation during IR, weaker antioxidant capacity, aggravated oxidative stress and enhanced IR injury (red arrows).

The histological features of NASH are vesicular steatosis and lobular hepatitis with necrosis or balloon degeneration and fibrosis (52, 53). According to the percentage of fat content in the liver, hepatic steatosis can be divided into mild, moderate and severe steatosis, and moderate to severe steatosis may lead to adverse outcomes after liver transplantation. However, views on the utilization of donor livers with moderate steatosis are not consistent (7). Steatosis hepatocytes are more sensitive to IR damage, possible reasons are the production of ROS, and more severe oxidative stress, inflammation, mitochondrial damage (**Figure 3**)

The mechanism underlying the pathogenesis and progression of NAFLD remains unclear, although the hypothesis of “multiple hits” is currently proposed. Insulin resistance, oxidative damage, stellate cell activation, fibrosis pathway activation, changes in the expression of adipokines, and many other sources of damage lead to the occurrence of NASH and liver cirrhosis (54). Excessive ROS generation and oxidative stress play an important role in the pathogenesis and development of NAFLD. In addition, mitochondrial oxidative stress is the main factor involved in the increased sensitivity of fatty liver to IR injury (55). Compared with healthy subjects, NAFLD patients have higher levels of ROS and lipid peroxidation products, lower levels of antioxidant enzymes [e.g. SOD and catalase (CAT)], and decreased levels of antioxidant compounds (e.g. GSH). The imbalance between the scavenging ability of antioxidants and ROS production led to oxidative stress (56, 57). In the pathogenesis of NAFLD, in order to eliminate excessive free fatty acids in the liver, mitochondria enhance β-oxidation and promote the production of ROS in the respiratory chain, leading to oxidative stress. Oxidative stress also aggravates lipid accumulation in hepatocytes, further promotes the production of ROS, and damages organelles, proteins, DNA, and lipids, resulting in a vicious cycle (58, 59). At the same time, the reaction of unfolded proteins in endoplasmic reticulum is enhanced, which also produces ROS (60).

**Figure 4:**
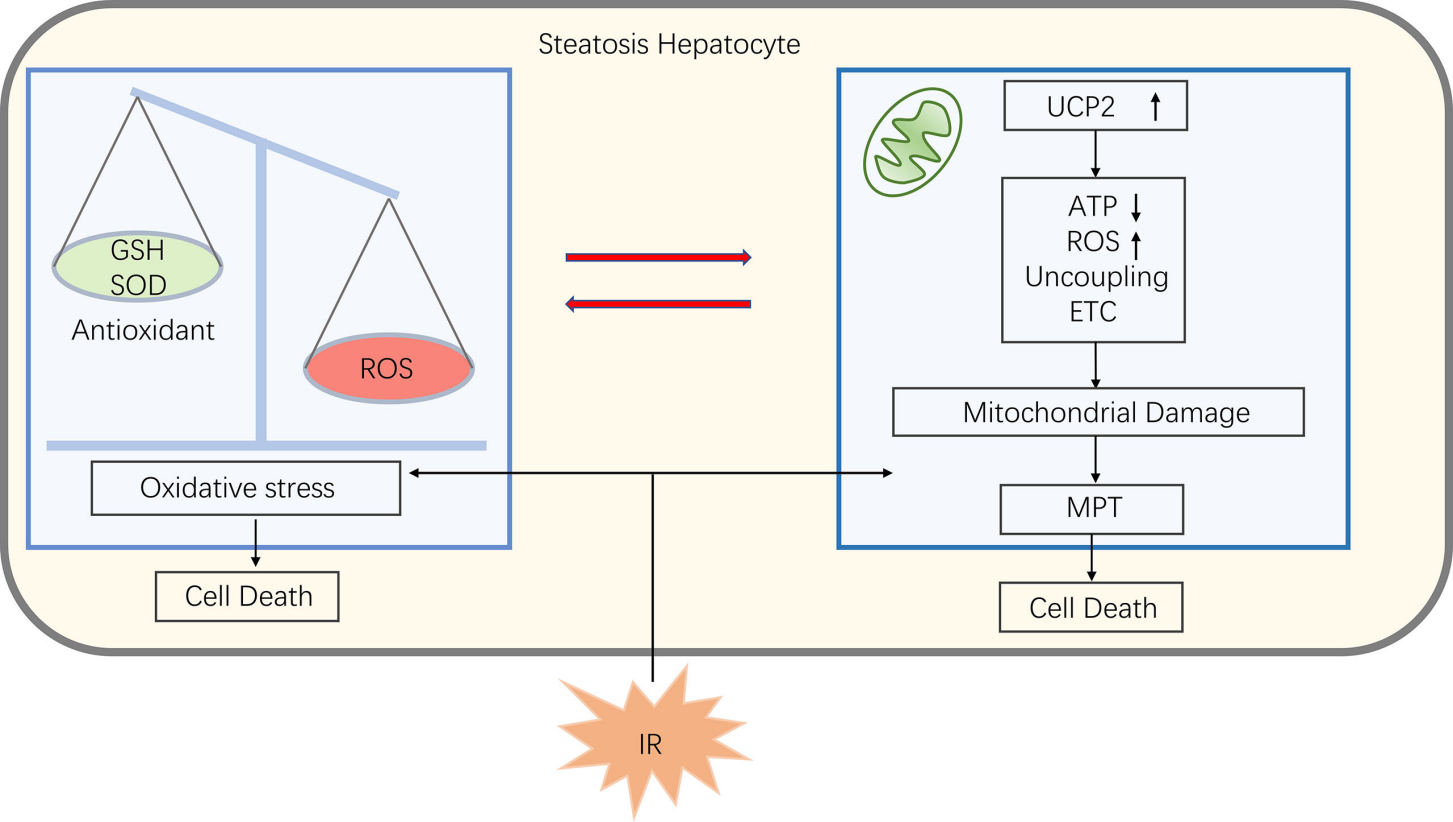
In steatosis hepatocyte, an imbalance of ROS and antioxidants leads to oxidative stress that damages mitochondria. Also oxidative stress promotes uncoupling protein 2 (UCP2) expression, which can uncouple electron transport chains (ETC), thereby reducing ATP synthesis and induce ROS production, further promoting oxidative stress. When fatty liver encounter IR, the mitochondria at this statute are more susceptible to mitochondrial permeability transitions (MPT), leading to cell death.

Some studies have found that mitochondrial dysfunction in liver tissue during NAFLD affects the liver lipid balance, promotes ROS production, lipid peroxidation as well as cytokine release, ultimately leading to cell death (61, 62). Damage to mitochondrial function in NAFLD makes mitochondria more susceptible to MPT and subsequently causing cell death when suffer from IR (25). Phospholipids on the mitochondrial membrane are oxidized, reducing fluidity and hindering the entry of GSH into the mitochondria, causing an imbalance between antioxidants and ROS, inducing oxidative stress, which makes increased expression of the uncoupling protein 2 (UCP2). UCP2 induces electron transport chains(ETC) uncoupling and thus reduces mitochondrial ATP synthesis (63). As a result, patients with NAFLD have lower reserves of ATP, and when the liver undergoing ischemia and hypoxia injury, ATP depletes faster, making cells more susceptible to necrosis and triggering an inflammatory response (25) (**Figure 4**).

**Figure 5:**
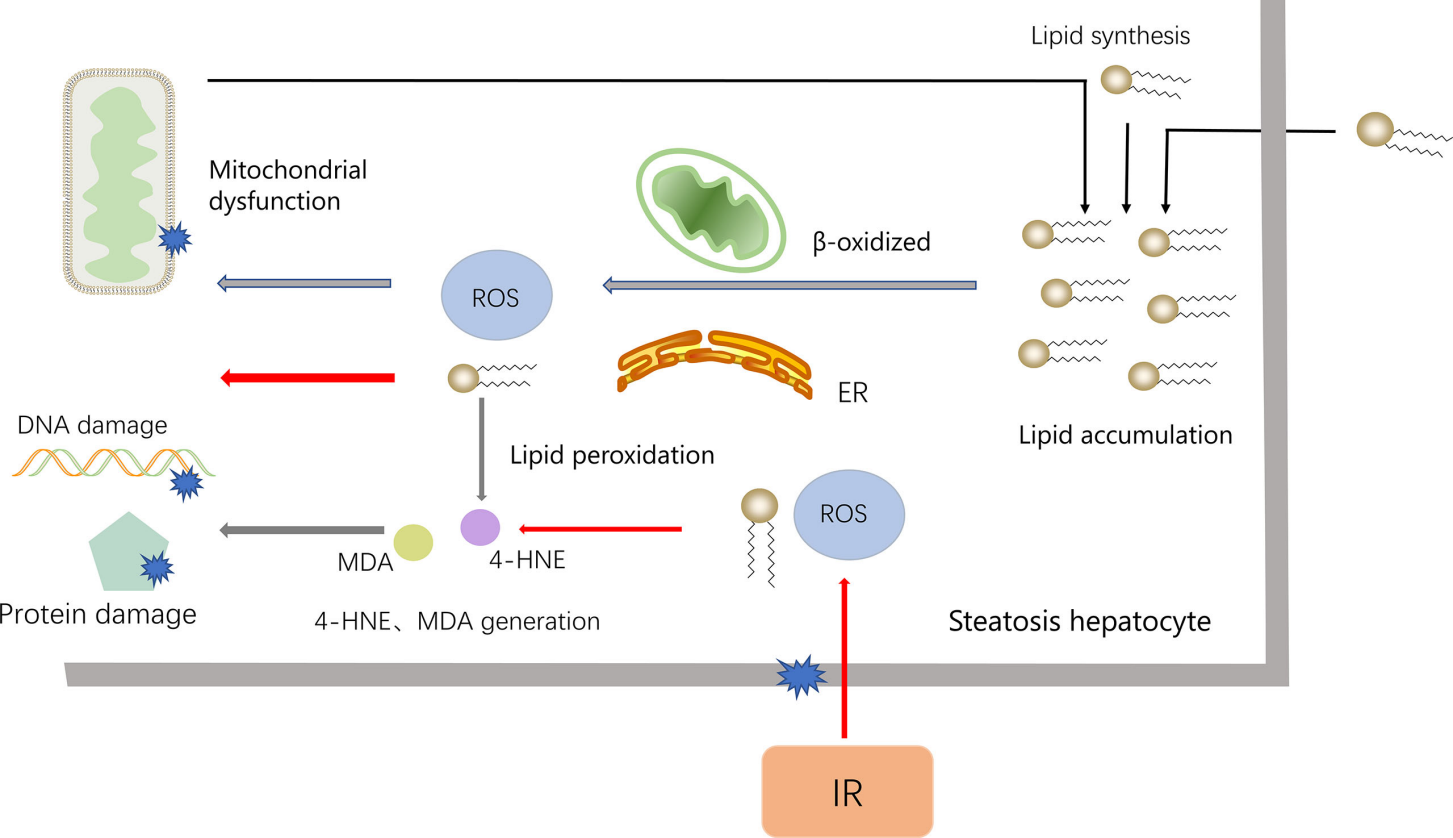
The accumulation of lipids is an important reason for the NAFLD. To process the accumulated lipids, hepatocytes increase mitochondrial β-oxidation and the activity of ER, which lead to the synthesis of ROS. ROS, in turn, damages the mitochondria, further leading to the accumulation of fatty acids (black arrows). When NAFLD encounters IR, ROS increased (red arrow), combined to accumulated fatty acids and release 4-HNE and MDA, which cause DNA and protein damage and worsen liver damage.

ROS binds to polyunsaturated fatty acids (PUFAs) to produce lipid peroxides. Unstable lipid peroxides are easily decomposed to active 4-hydroxy-2-nonenal (4-HNE) and Malondialdehyde (MDA), causing damage to cells (56, 64). In fatty liver IR, lipids are most vulnerable to ROS attack. However, NAFLD itself contains the accumulation of fatty acids and extensive lipid peroxidation, which makes the fatty liver IR injury more serious (54). In the pathogenesis of NALFD, ROS also attack proteins, especially antioxidant enzymes, and the antioxidant capacity is weakened after oxidation. Furthermore, in NAFLD, GSH is chronically consumed, and the combination of lipid peroxidation products with GSH also consumes GSH (65) (**Figure 5**).

Peroxisome proliferator-activated receptor-α (PPAR-α) and Peroxisome proliferator-activated receptor-γ (PPAR-γ) are important regulators of IR damage in steatotic liver (55). The PPAR-γ agonist WY-14643 reduces steatosis and the IR injury in mouse liver (66). In addition, studies have shown that pioglitazone-activated PPAR-γ attenuates liver injury induced by IR. In contrast, mice with a significantly decreased expression of PPAR-γ showed relatively severe liver injury (67, 68). PGC-1α regulates antioxidants *in vivo*, and its overexpression can significantly reduce ROS in IR, whereas knockout of PGC-1α leads to a further increase in the intracellular ROS level (69). ROS is produced during NAFLD, which consumes antioxidants and damages mitochondria. On this basis, when IR occurs, more ROS are produced, more antioxidants are exhausted, and oxidative stress and damage are exacerbated.

## Possible Strategies for Treating IR

Given the current high proportion of donor hepatic steatosis and the increase in the number of fatty liver patients on the liver transplant waiting list, improving the availability of grafts and reducing the risk of post-transplant dysfunction has become a hot topic. Numerous studies have been conducted, including the treatment of potential donors for transplantation, as well as treatment during transplantation, thereby improving the success rate of transplantation. Here, we summarize some of the treatments for some antioxidant therapies during transplantation (**Table 1**) and for potential donors diagnosed with NAFLD/NASH.

**Table 1:** Possible treatment or drugs on alleviating IR injury and mechanism.

Reference	Treatment or drugs	population	Effects	
(70)	Ischemic Preconditioning	Human	ALT, AST↓, caspase 3↓	
(71)	Iischemic Preconditioning	Zucker rat	Xanthin→XOD↓, MDA↓, P38MAPK↓, JNK↓, HSP72↑, HO-1↑	
(72)	Isoflurane Preconditioning	Mice	AST, ALT, LDH↓, MiR-142↑, HMGB1↓, TLR4/NF-κB pathway activation↓	
(39)	Sevoflurane Preconditioning	Rat	HO-1↑, AST, ALT↓, TNF-α↓, MDA↓, MPO↓	
(73)	Gastrodin Preconditioning	Mice	AST, ATL↓, MDA↓, SOD↑, IL-6↓, TNF-α↓, Nrf2↑, p38MAPK↑	
(74)	Vitamin E succinate Preconditioning	Mice	UCP2↓, ATP↑, GSH↑	
(75)	CDP-choline Preconditioning	rat	AST, ALT↓, ROS↓, caspase-3↓	
(76)	Lipid nanoparticles	mice	EC-SOD↑, catalase↑, H_2_O_2_↑, GSH↑, MDA↓	
(77, 78)	N-Acetylcysteine (NAC)	mice/rabbit	AST↓, IL-1β↓, TGF-β1↓, bile flow↑, ROS, RNS↓	
(79)	Melatonin	Zucker rat	ATL, AST↓, MDA↓, mRNA expressions of iNOS and eNOS↓, Nox metabolite level↓, GSH/GSSG↑, expression of Bax, Bad, AIF↑, caspase9 activity↓	
(80)	Aloin	mice	ALT, AST↓, GSH↑, SOD↑, MDA↓, ROS↓, IL-6↓, TNF-α↓, IL-10↑, caspase 3↓, Bcl-2↑, Bax↓, inhibit the TLR4/MyD88/NF-κB siginal pathway	
(42)	Ginsenoside Rg1	rat	AST, ALT↓, caspase 3↓, caspase 9↓, CypD↓
(81)	Tea polyphenols	mice	AST, ALT↓, GSH/GSSG↑, iNOS↑, Bax↓, cytochrome c↓, caspase 3↓	
(82)	Grape seed proanthocyanidins	rat	ALT, AST↓, TGF-β1↑, IL-10↑, TNF-α↓, IL-6↓, SOD↑, MDA↓, procaspase-12↑, GRP78↑, IRE-1↓, ATF-4↓, NF-κB↓, downregulates IRE-1/NF-κb and ATF-4/CHOP signal	
(83)	Irisin	mice	ALT, AST↓, LDH↓, caspase 3↓, MPO↓, CIRP↓, TNF-α↓, DRP-1↓, FIS-1↓, mtDNA copy↑, PGC-1α↑, TFAM↑, MDA↓, Gpx↓, SOD↑, UCP-2↑, restrain mitochondrial fission, promote mitochondrial biogenesis	
(84)	CoPP	Zucker rat	bile production↑, portal blood flow↑, sGOT↓, HO-1↓	
(67)	Pioglitazone	mice	ALT, AST, LDH↓, TNF-α↓, IL-1β↓, MCP-1↓, MIP-2↓,IP-10↓, iNOS↓, eNOS↓, MPO↓, caspase 3↓	
(85)	Shikonin	mice	ALT, AST↓, IL-1β↓, TNF-α↓, IL-6↓, Bcl-2↑, Bax↓, caspase 3↓, caspase 9↓, Beclin-1↓, LC3↓, PI3K↑, p-Akt↑	
(86)	Vitamin D	mice	ALT, AST↓, MDA↓, Mn-SOD↑, GSH/GSSG↑, catalase↑, TNF-α↓, IL-6↓, IL-2↓, MPO↓, LC3II↑, Beclin-1↑, ATG-7↑, PTEN↓, pAkt↑, mTOR↓	

↑means increase, ↓means decrease.

### Preconditioning

A common anti-IR approach is ischemic preconditioning (IPC), the process of transient IR before a long period of ischemia. An experimental model showed that liver IPC can protect the liver from the adverse effects of IR, and clinical practice has also demonstrated the protective effect of IPC on steatotic liver (70, 87). IPC reduce the production of ROS by inducing the production of NO, reducing the accumulation of xanthine during ischemia, and preventing the conversion of XDH to XOD (88). IPC also regulates inflammatory factors, activates the PPAR-α signaling pathway, and reduces the production of ROS, thereby reducing oxidative stress, mitochondrial dysfunction, and the activation of neutrophils and KCs (9). IPC over-induced heme oxygenase-1 (HO-1) can protect fatty liver from IR injury, and the protective effect of IPC is weakened after using HO-1 inhibitors (71). Studies have shown that the protective effect of IPC on steatotic liver IR injury is related to the activation of AMPK. AMPK activation reduces the further consumption of ATP, promotes NO synthesis, inhibits the NF-κB signaling pathway, and reduces lipid peroxidation and liver cell damage (55).

In addition, simulating the effect of IPC by regulating the internal and external components of cells *via* drugs is called pharmacological preconditioning (31). Pretreatment with volatile anesthesia, such as isoflurane and sevoflurane, reduces IR injury in mice (39, 72). Sevoflurane reportedly inhibits the expression of HMGB1 by up-regulating the expression of miR-142 in IR mice and inhibits the activation of the TLR4/NF-κB inflammatory pathway, thereby reducing oxidative stress and liver damage (39). Preconditioning with gastrodin, vitamin E succinate, CDP-choline, and other drugs protects the liver from IR injury (73–75).

### Anti-Oxidants

In fatty liver, the SOD activity and GSH levels decrease. When such livers are exposed to IR, these parameters decrease even further, leading to a sharp increase in ROS and thereby promoting an imbalance between oxidation and reduction, leading to oxidative stress. Therefore, antioxidants play an important role in reducing fatty liver IR (72). Common endogenous antioxidant enzymes include SOD, CAT, GSH, and vitamin C. SOD catalyzes the superoxide anion and water to form H_2_O_2_ and remove ROS (89). There are three isozymes of SOD: copper/zinc SOD (Cu/Zn-SOD), manganese SOD (Mn-SOD), and extracellular SOD (EC-SOD). In IR, the inactivation of the MnSOD function weakens the antioxidant capacity of NAFLD and affects the viability of hepatocytes (90). CAT is a H_2_O_2_ scavenger that plays an additional scavenging role when SOD is insufficient to remove ROS. Lipid nanoparticle-mediated antioxidant gene delivery significantly increases the expression of human EC-SOD and CAT genes in the liver, thus reducing IR injury (76).

The application of antioxidants also reduces the oxidative stress of liver IR. At present, the studies of antioxidants to alleviate ischemia and reperfusion damage in animal models is intense with fewer studies of antioxidants in human ischemia and reperfusion damage. GSH is a substrate of Gpx and decomposes the precursor molecule of H_2_O_2_. N-Acetylcysteine (NAC) has been shown to increase the activity of reduced GTH-related enzymes, reduce ROS, and improve IR injury in fatty liver (77, 78). The administration of postoperative or peri-operative NAC in patients undergoing hepatic resection did not achieve great complication prevention results (91, 92). In another study, the use of NAC during donor liver collection significantly improves graft survival and post-transplant outcomes (93). Existing antioxidant studies of human ischemia-reperfusion injury are rarely assessed for antioxidant capacity. Melatonin alleviates IR injury in rats with fatty liver degeneration by strengthening the clearance of ROS and NOS, restoring the mitochondrial function, reducing the expression of pro-apoptotic genes, and reducing oxidative stress (79). In a randomized controlled double-blind pilot clinical trial, patients received preoperative care of high-dose (50 mg/Kg) melatonin, exhibiting less non-infectious complications ICU and total hospital stay, the authors speculate that antioxidant capacity is a possible cause (94). The increase in the ratio of ω6/ω3 plays an important role in NAFLD, and the NAFLD patients suffer from less damage when the ratio decreases. ω3 polyunsaturated fatty acids (PUFA) plays good anti-inflammatory activity, while reducing the fat content of the liver (95, 96). In animal renal ischemia-reperfusion models, ω3PUFA has been reported to reduce damage through antioxidant action (97). Mice consuming a omega-3PUFA-containing diet were less sensitive to liver ischemia reperfusion injury (98). Previous studies have shown that postoperative use of omega-3 PUFA parenteral nutrition has some benefit in postoperative liver patients (95, 99). In contrast to previous animal experiments reported to reduce liver damage and improve liver regeneration a recent study showed that perioperatively intravenous omega-3PUFA achieve no improvement on complications (100).

In addition, some antioxidants have been shown in animal models to reduce oxidative stress. Aloin increases the SOD activity and GSH level, reduces oxidative stress and inflammation, and alleviates liver IR injury (80). Ginsenoside Rg1 plays a protective role by inhibiting the expression of apoptosis-related proteins and down-regulating inflammatory mediators and antioxidation (42, 101). Tea polyphenols have strong antioxidant properties. The use of tea polyphenols increases the level of GSH, inhibits oxidative stress, and alleviates IR in mice (81). Grape seed proanthocyanidins protects against IR injury through antioxidant, anti-inflammatory, and anti-apoptotic effects (82). Irisin can relieve oxidative stress by mediating the expression of coupling protein 2 and ameliorate IR through its anti-apoptotic effect (83). Curcumin increases animal survival after transplantation by playing a protective role against inflammation and oxidative stress (102, 103).

Some endogenous genes or gene products have been proven to exert protective effects in IR injury. Nrf-2 is a transcription factor that is sensitive to oxidative stress and positively regulates the basic and induced expression of a large number of cytoprotective genes (104). In liver IR, the regulation of the keap1-Nrf2-ARE pathway promotes the gene expression of antioxidant enzymes, such as SOD, GTH peroxidase, CAT, and HO-1, thereby playing a protective role (105, 106). Under oxidative stress, Nrf2 is activated, and the expression of HO-1 is up-regulated. HO-1, with NADPH as a cofactor, catalyzes the oxidative degradation of heme to the antioxidants biliverdin, carbon monoxide, and iron, and exerts the effect of scavenging ROS and anti-inflammation (107, 108). The up-regulation of HO-1 expression by HO-1 inducers, such as cobalt protoporphyrin and adenovirus HO-1, alleviates steatotic liver IR injury in rats (84). ROS activates the PI3K/Akt pathway, and the activation of Akt inactivates p53 and other pro-apoptotic proteins, thereby increasing the expression of anti-apoptotic proteins and inhibiting cell apoptosis. Shikonin treatment reduce IR injury by activating PI3K/Akt (85). Vitamin D treatment of mice was shown to reduce ROS and down-regulate the PTEN-activated PI3K/Akt pathway, thus regulating autophagy and reducing injury (86).

### Treatment for NAFLD

**Figure 6:**
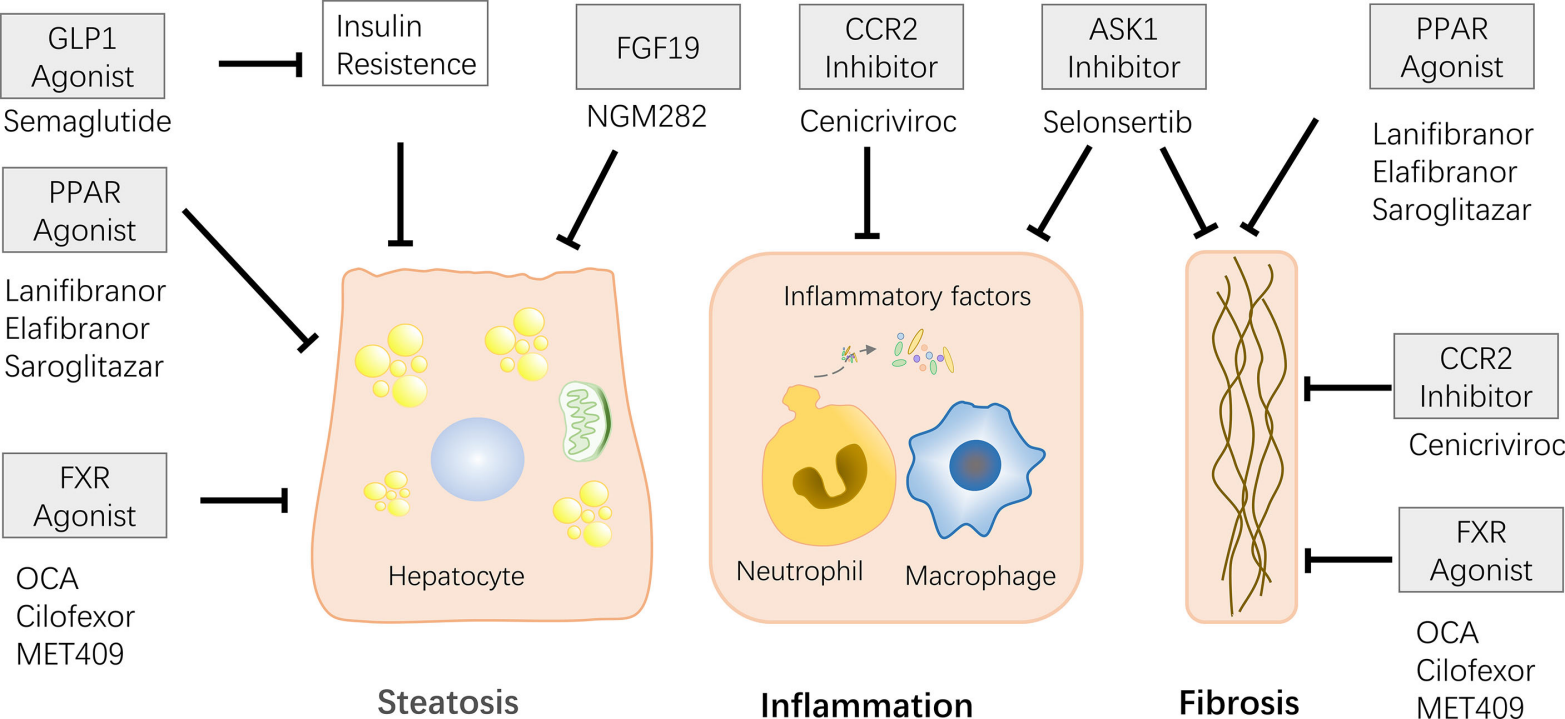
Some of the drugs currently in clinical trials and their effects are described. Effects include relieving steatosis, inflammatory response, and liver fibrosis. FXR agonists and PPAR agonists can alleviate steatosis and liver fibrosis. CCR2/5 and ASK1 inhibitors play role on anti-inflammatory and anti-fibrotic. FGF19 has the ability to relieve steatosis. FLP-I relieves steatosis by relieving insulin resistance.

Treatment of potential donors prior to living donor transplantation (LDLT) could alleviate steatosis, increase donor availability to some extent, and reduce IR damage. The common methods include diet, exercise, and drugs (**Figure 6**). Physical activity and dietary restrictions are routine treatments for patients with NAFLD. It is reported that performing living liver transplantation with diet-treated donors is feasible (109). Short-term weight loss through diet combined with exercise can alleviate the degree of fat infiltration of fatty liver donors, and most donor who receive weight loss successfully complete LDLT (110–113).

The only current study of preoperative short-term drug therapy for donors was benzalabet (an activator of PPARα and PPARβ/δ). In a study of preoperative short-term treatment of donors, they were treated with a protein-rich (1000 kcal/day) diet, exercise (600 kcal/day) and benzabrate (400 mg/day) for 2-8 weeks before surgery, the authors observed a reduction in steatosis, no significant complications after surgery, and the transplanted grafts and donors showed good liver function (114).

PPAR is a hot therapeutic target, has been reported great capacity of reduce steatosis, inflammation and liver fibrosis (115–117). PPAR-γ has a protective effect on liver tissue in IR. IR injury can be alleviated by increasing the levels of SOD and hydrogen peroxide hydrolase and reducing IR injury using pioglitazone, a PPAR-γ agonist (67). Other PPAR agonists have also reported surprising results in clinical trials. Lanifibranor, a pan PPAR agonist that activates three PPARs, lowered the SAF-A score and relieved NASH in a recently published Phase 2b randomized, double-blind, placebo-controlled trial (115). Elafibranor (PPAR-α/δ agonist) is reported to alleviate histologic changes and improve metabolism in NASH patients (116). The phase 2 trial of Saroglitazar (a PPAR-α/γ agonist) reported the effect of remission of insulin resistance, atherosclerotic dyslipidemia, and ALT, LCF parameters in patients with NAFLD/NASH (117).

Farnesoid X receptor (FXR) is a member of the nuclear receptor superfamily that regulates bile acid metabolism, lipid metabolism, and glucose metabolism. Elevated bile acid levels could inhibit bile acid synthesis by activating FXR-SHP-SREBP1c (118, 119). FXR is now considered as new drug target, and clinical trials of some FXR agonists are underway. Obeticholic acid (OCA), a semi-synthetic derivative of primary human bile acid goose deoxycholic acid, works in animal models through reduce insulin resistance and hepatic steatosis. Previous studies have shown that after 6 weeks of treatment with 25/50 mg OCA, there was a decrease in liver inflammation and fibrosis markers in patients with type 2 diabetes and non-alcoholic fatty liver disease (120). Subsequent Phase 3 experiments on the treatment of NASH with OCA confirmed that it reduces NASH fibrosis and disease activity indicators (121). However, a large number of patients have experienced the side effect of itching after taking OCA, thus other FXR agonists without bile acids have been developed. MET409 is a novel FXR agonist without bile acid structure, has been reported to reduce liver fat content in NASH patients (122). Clinical trials of Cilofexor (nonsteroidal FXR agonist) have also reported similar results (123).

When FXR is activated by bile acids, fibroblast growth factor (FGF) 15 is induced, play the role of inhibiting the synthesis of bile acids through the down regulate the transcription of cholesterol 7α-hydroxylase (Cyp7a1). FGF19 is a human homolog of FGF15, is also regulated by the level of bile acids (124, 125). FGF19 analogues NGM282 and Aldafermin have been designed as novel therapeutics and are currently in clinical trials. The phase 2 trial of NGM282 confirmed that NGM282 induce a decrease in liver fat content in NASH patients (126). Similarly, phase 2 trials of Aldafermin confirmed its effects on reducing liver fat (127).

New drugs for other targets have also been designed, including targeting C-C motif chemokine receptor (CCR)2-CCR5, apoptosis signaling protease-1 (ASK-1), glucagon-like peptide (GLP)-1 and etc. CCR2/5 improve liver fibrosis by activating inflammatory signals and immune cell infiltration, causing liver damage (128). Cenicriviroc (CVC), an oral dual CCR2/CCR5 antagonist, the recent Phase 3 clinical trials surprisingly reported potent anti-inflammatory and antifibrotic activity (129). Oxidative stress activates the ASK1-Jun pathway, leading to increased liver inflammation and fibrosis. Selonsertib, a selective inhibitor of ASK1, was reported to reduce liver fibrosis in patients with NASH and stage 2-3 fibrosis in previously published phase 2 experiments. However, the recently published phase 3 experiment proved that there was no mitigating effect on fibrosis in patients with NASH-induced bridging fibrosis or compensated cirrhosis (130). Glucagon-like peptide (GLP)-1 stimulate insulin secretion and inhibit the secretion of glucagon, also has a certain effect of relieving NASH, authors observed that subcutaneous injection of Semaglutide in NASH patients could achieve NASH regression (131).

## Conclusion and Future Directions

IR causes the adverse outcome of liver transplantation, and steatosis liver is more sensitive to IR, therefore, the steatosis liver transplantation is more likely to get the adverse outcome, which brings challenges to clinical work. The ROS production in non-alcoholic fatty liver is substantial, and IR further promotes ROS production, oxidative stress, and inflammation, thus exacerbating the condition. Preconditioning and certain antioxidants can reduce the IR injury seen in fatty liver in mice. Treatment of potential patients with fatty liver can increase transplant safety. However, the mechanisms underlying IR and the generation of non-alcoholic fatty liver itself are unclear. The rapid increase in fatty liver patients requires further research into the mechanism underlying fatty liver IR injury. At the same time, the drug treatment of NAFLD is also expected. In addition, most studies on antioxidant therapy are focused on IR, and clinical data are lacking. Therefore, further research on the treatment of fatty liver IR is needed.

## Author Contributions

All of the authors contributed to the writing and editing of the manuscript and approved the submitted version.

## Funding

This work was supported in part by the Medical Science and Technology Project of Zhejiang Province (2021PY083), Program of Taizhou Science and Technology Grant (20ywb29), Major Research Program of Taizhou Enze Medical Center Grant (19EZZDA2), Open Project Program of Key Laboratory of Minimally Invasive Techniques & Rapid Rehabilitation of Digestive System Tumor of Zhejiang Province (21SZDSYS01, 21SZDSYS09) and Key Technology Research and Development Program of Zhejiang Province (2019C03040).

## Conflict of Interest

The authors declare that the research was conducted in the absence of any commercial or financial relationships that could be construed as a potential conflict of interest.

## Publisher’s Note

All claims expressed in this article are solely those of the authors and do not necessarily represent those of their affiliated organizations, or those of the publisher, the editors and the reviewers. Any product that may be evaluated in this article, or claim that may be made by its manufacturer, is not guaranteed or endorsed by the publisher.
